# From structural repair to biomechanical compatibility: Aortic stiffness after open and endovascular aortic repair

**DOI:** 10.1113/EP092443

**Published:** 2026-10-26

**Authors:** Thurkga Moothathamby, Matti Jubouri, Fatima Kayali, Sakshi Roy, Subham Roy, Idhrees Mohammed, Ian M. Williams, Damian M. Bailey, Mohamad Bashir

**Affiliations:** ^1^ Charing Cross Hospital, Imperial College Healthcare NHS Foundation Trust London UK; ^2^ Queen Elizabeth Hospital, University Hospitals Birmingham NHS Foundation Trust Birmingham UK; ^3^ Department of General Surgery Royal Liverpool University Hospital Liverpool UK; ^4^ School of Medicine Queen's University Belfast Belfast UK; ^5^ Hull York Medical School University of York York UK; ^6^ Institute of Cardiac and Aortic Disorders SRM Institutes for Medical Science (SIMS Hospital) Chennai Tamil Nadu India; ^7^ Department of Vascular Surgery University Hospital of Wales Cardiff UK; ^8^ Neurovascular Research Laboratory, Faculty of Life Sciences and Education University of South Wales Pontypridd UK

**Keywords:** aortic stiffness, carotid‐femoral pulse‐wave velocity, compliance mismatch, endovascular aneurysm repair, open surgical repair, thoracic endovascular aortic repair

## Abstract

Evaluation of aortic repair has traditionally prioritised perioperative safety, exclusion of the diseased segment and durability. As endovascular treatment expands to younger patients, attention is turning towards the biomechanical compatibility of prosthetic grafts with the native aorta. Native aortic compliance supports Windkessel function, whereas grafts create a compliance transition that may accelerate pulse‐wave transmission, alter wave reflection and increase left‐ventricular load. This narrative review distinguishes established evidence from emerging concepts after open surgical repair, endovascular aneurysm repair and thoracic endovascular aortic repair. Carotid‐femoral pulse‐wave velocity (cfPWV) is the accepted reference non‐invasive measure of regional aortic stiffness; cardiovascular magnetic resonance (CMR)‐derived segmental pulse‐wave velocity and distensibility provide complementary information. Brachial‐ankle pulse‐wave velocity, the cardio‐ankle vascular index, and augmentation index assess different territories or haemodynamic properties and are not interchangeable with cfPWV. Left‐ventricular strain and mass are downstream cardiac outcomes. Clinical evidence generally reports increases in measured arterial‐stiffness indices after endovascular repair, whereas findings after open repair are heterogeneous. Treatment length, landing‐zone location, oversizing, anatomy and baseline vascular phenotype may be as important as graft fabric or stent‐frame category. Conflicting comparisons of polyester and expanded polytetrafluoroethylene arise from small, non‐randomised studies using non‐equivalent endpoints and do not support a reliable ranking of commercial devices. Emerging 4D‐flow CMR, simulation and compliant graft materials illustrate a shift from structural exclusion alone towards compliance‐aware repair, but evidence of improved outcomes remains insufficient. Future studies should use standardised longitudinal stiffness, blood‐pressure and cardiac assessments and relate biomechanical changes to hypertension, ventricular remodelling and cardiovascular outcomes.

## Abstract (highlights)

- **What is the topic of this review?**
  This review examines how open and endovascular repair alter aortic mechanics, pulse‐wave transmission and cardiac load.
- **What advances does it highlight?**
  Carotid‐femoral pulse‐wave velocity remains the reference measure of regional aortic stiffness; segmental cardiovascular magnetic resonance (CMR), 4D‐flow CMR and modelling add complementary mechanistic insight. Stiffness generally increases after endovascular aneurysm repair and thoracic endovascular aortic repair; open‐repair findings vary with anatomy, graft length, vascular phenotype, measurement pathway and follow‐up. Compliance‐aware planning and biomimetic grafts are emerging but investigational. Evidence does not support device selection based solely on arterial stiffness.

## 1 INTRODUCTION

Arterial stiffness has been investigated for several decades and is an established marker of cardiovascular ageing and risk. Carotid‐femoral pulse‐wave velocity (cfPWV) is the accepted reference non‐invasive measure of regional aortic stiffness. Greater cfPWV reflects faster propagation of the pressure wave through a less compliant arterial pathway and is associated with cardiovascular events and mortality (Ben‐Shlomo et al., 2014; Laurent et al., 2001; Van Bortel et al., 2012). However, cfPWV should be distinguished from measurements incorporating substantial peripheral arterial segments, such as brachial‐ankle pulse wave velocity (baPWV), and from local imaging‐derived measurements of aortic distensibility or pulse‐wave velocity. Endothelial‐function measurements and cardiac indices such as left‐ventricular strain are physiologically relevant but are not measures of arterial stiffness.

The native aorta stores energy during systole and releases it during diastole, moderating pulsatile pressure and supporting coronary and peripheral perfusion. Aortic aneurysm, dissection and ageing alter this function before intervention. Open surgical repair (OSR) and endovascular stent‐grafting further modify the mechanical environment by introducing prosthetic segments that are generally less compliant than the adjacent native aorta. Consequently, post‐repair measurements reflect the combined effects of the underlying disease, the treated segment, the prosthesis and the remaining arterial pathway.

Historically, the success of aortic repair has appropriately been judged by survival, exclusion of the diseased aorta, graft patency, prevention of disease progression or rupture, and repair durability. These remain the principal clinical objectives. The emerging question is whether the biomechanical transition created by repair has additional long‐term consequences, particularly as endovascular treatment is offered to younger patients and increasingly extensive aortic segments are covered. Potential consequences include accelerated pulse‐wave transmission, altered wave reflection, greater pulsatile blood pressure and increased left‐ventricular afterload. Recent segmental imaging, patient‐specific modelling and experimental compliant grafts have therefore shifted research from describing postoperative stiffness alone towards investigating biomechanical compatibility.

This narrative review does not attempt to rank stiffness measures or commercially available grafts. Instead, it examines what is established about changes in arterial stiffness after open and endovascular repair; why findings differ among studies; how repair‐related biomechanical changes may relate to blood pressure and cardiac remodelling; and which emerging technologies and standardised research methods could move the field forward. The narrative review encompasses thoracic, abdominal and thoracoabdominal repair, while recognising that dedicated evidence following thoracoabdominal and fenestrated or branched endovascular repair remains sparse.

## 2 METHODS

This narrative review was informed by searches of multiple electronic databases including PubMed, Ovid MEDLINE, Embase and Scopus from database inception until May 2025. The literature was updated through September 2026 using PubMed and citation tracking. Search terms combined concepts relating to arterial or aortic stiffness, pulse‐wave velocity, aortic compliance, OSR, endovascular aneurysm repair (EVAR), thoracic endovascular aortic repair (TEVAR), graft materials, stent‐graft design, central haemodynamics and left‐ventricular remodelling. Reference lists of relevant reviews and relevant studies were examined to identify additional publications.

Priority was given to clinical studies evaluating patients before and after aortic repair or directly comparing open and endovascular approaches. Experimental, *ex vivo* and computational studies were included where they provided mechanistic evidence concerning compliance mismatch or emerging graft technologies and are identified as such throughout. Because this was a narrative rather than systematic review, no protocol registration, formal risk‐of‐bias assessment or quantitative meta‐analysis was undertaken. Conclusions were calibrated according to study design, sample size, measurement pathway and consistency across studies.

Ethical approval and consent to participate were not required because this article is a narrative review of previously published literature. Consent for publication was not applicable.

## 3 FINDINGS

### 3.1 Non‐invasive methods for aortic stiffness assessment

Arterial stiffness cannot be represented by a single interchangeable measurement. Pulse‐wave velocity describes the speed at which the arterial pressure wave travels between two defined recording sites; consequently, the anatomical pathway must be stated whenever results are reported. Higher pulse‐wave velocity generally indicates lower arterial compliance, although the measured value is also influenced by blood pressure, heart rate, age and the method used to estimate travelled distance.

cfPWV is the accepted reference non‐invasive measure of regional aortic stiffness. It principally reflects the stiffness of the descending thoracic and abdominal aortic pathway and has established prognostic value. Its limitations, including dependence on surface‐distance estimation and the inability to localise stiffness changes to an individual aortic segment, do not invalidate it as a measure of aortic stiffness. Instead, these limitations define the circumstances in which complementary segmental measurements may be informative (Townsend et al., 2015; Van Bortel et al., 2012). Standardisation of patient preparation, measurement distance, device validation and concurrent blood‐pressure reporting is essential when comparing serial or between‐study values; values generated by different tonometric and oscillometric systems should not be assumed to be interchangeable (Del Giorno et al., 2021).

Cardiovascular magnetic resonance (CMR) can measure aortic distensibility, strain and pulse‐wave velocity within specified thoracic or abdominal segments. These measurements may help distinguish the mechanical behaviour of the graft, the immediately adjacent native aorta and more remote aortic segments. Phase‐contrast and 4D‐flow techniques can additionally characterise flow propagation and secondary‐flow patterns. CMR should therefore be presented as complementary to cfPWV rather than as a more accurate replacement for it (Cavalcante et al., 2011; Redheuil et al., 2010; Swoboda et al., 2018). Echocardiography can estimate local aortic strain or distensibility when suitable imaging windows are available, but left‐ventricular global longitudinal strain (GLS), mass and diastolic indices remain downstream cardiac outcomes.

Other non‐invasive indices measure different vascular territories or haemodynamic properties. baPWV includes substantial peripheral arterial segments and should not be described as a direct measure of isolated aortic stiffness. The cardio‐ankle vascular index (CAVI) similarly reflects the arterial pathway from the origin of the aorta to the ankle and is designed to reduce, but does not completely eliminate, dependence on blood pressure at the time of measurement (Saiki et al., 2016). The augmentation index reflects the timing and magnitude of wave reflection and is influenced by heart rate, vascular tone, height and peripheral reflection sites; it is not interchangeable with cfPWV.

Earlier clinical studies generally relied on regional pulse‐wave or wave‐reflection measurements. Emerging imaging approaches add spatial resolution by characterising stiffness within and around a treated segment. The developing methodological direction is therefore to combine a standardised reference measure such as cfPWV with carefully selected local imaging and cardiac outcomes, rather than to seek a replacement for cfPWV.

### 3.2 What does endovascular aortic repair offer?

The established structural objective of endovascular repair is to exclude or stabilise the diseased aortic segment while reducing the physiological burden of open surgery. From a biomechanical perspective, however, deployment of a stent‐graft introduces a relatively stiff prosthetic segment within a compliant native arterial pathway. The resulting compliance transition may accelerate pulse‐wave transmission and alter wave reflection, central pressure and the mechanical loading of adjacent aortic segments. These effects do not diminish the established clinical benefits of EVAR or TEVAR, but they have become increasingly relevant as endovascular treatment is used in younger patients and across longer aortic segments.

Clinical studies of infrarenal EVAR have generally reported postoperative increases in arterial‐stiffness indices, although the magnitude and timing vary. Kadoglou et al. (2012) reported that cfPWV increased from 13.11 ± 3.57 to 16.41 ± 2.33 m/s after EVAR in 48 patients. Moulakakis et al. (2015) similarly identified increases in cfPWV and wave‐reflection indices after endograft implantation. More recently, Bellotti et al. (2026) prospectively evaluated 38 patients immediately before and seven days after elective EVAR: median within‐person cfPWV increased by 1.0 m/s, while the subendocardial viability ratio decreased; graft length correlated with the change in cfPWV. These short‐term findings support an acute haemodynamic effect but do not establish persistence or clinical consequence.

Synthesis of the available evidence also requires caution. Bissacco et al. (2022) found that endovascular repair was generally associated with increased arterial stiffness, but emphasised substantial heterogeneity in measurement pathways, timing and procedural characteristics. A later meta‐analysis of 28 studies and 2227 participants reported an overall increase in pulse‐wave velocity after endovascular repair, with similar directional findings after EVAR and TEVAR, but heterogeneity remained high, particularly in the abdominal aorta (*I* ^2^  =  90%) (Chen et al., 2026). Thus, the literature supports an overall signal of increased stiffness without establishing a uniform effect size for every patient, pathology or device. The principal clinical studies are summarised in Table 1.

**TABLE 1:** Principal clinical evidence after endovascular aortic repair.

Study	Population and repair	Exact assessment and timing	Principal finding	Interpretation/limitation
Kadoglou et al. (2012)	48; infrarenal EVAR	cfPWV; pre‐ and post‐repair	13.11 ± 3.57 to 16.41 ± 2.33 m/s; significant increase	Small single‐centre cohort; regional measure
Takeda et al. (2014)	40; EVAR/TEVAR	baPWV; baseline, 1 week and 1 year	baPWV and LV mass/left‐atrial volume indices increased	Mixed repairs; baPWV includes peripheral arteries
Hori et al. (2020)	86; TEVAR for arch aneurysm	baPWV; perioperative and median 32‐month follow‐up	Progressive increase with exoskeleton; no significant change with endoskeleton	Non‐randomised device comparison
Yamashita et al. (2021)	64; TEVAR	baPWV; pre‐ and post‐repair	1851 ± 392 to 2047 ± 479 cm/s; 58% increased ≥100 cm/s	Peripheral pathway; haemodynamic confounding
Hori et al. (2021)	183; TEVAR	baPWV; measurements within 1 year	Treatment length and device selection were associated with change; treatment site was not	Retrospective; device selection non‐random
Guala et al. (2024)	26 TEVAR for BTAI; 37 matched controls	cfPWV, 24‐h BP and CMR; mean 10‐year follow‐up	65% hypertension; 54% abnormal dipping; 23% high cfPWV	Small trauma cohort; observational associations
Bellotti et al. (2026)	38; elective infrarenal EVAR	cfPWV and SEVR; <72 h before and 7 days after	Median cfPWV +1.0 m/s; SEVR ‐15.1%; graft length correlated with cfPWV change	Preliminary short‐term findings
Hauguel et al. (2026)	14 patients; 15 descending TEVAR procedures	4D‐flow CMR and segmental PWV; pre‐ and approximately 3 months post	Stented‐segment stiffening with distinct upstream flow and PWV changes	Small mechanistic cohort; segment‐specific endpoint

*Note*: Different pathways and methods are not directly interchangeable. Abbreviations: baPWV, brachial‐ankle pulse‐wave velocity; BP, blood pressure; BTAI, blunt thoracic aortic injury; cfPWV, carotid‐femoral pulse‐wave velocity; CMR, cardiovascular magnetic resonance; EVAR, endovascular aneurysm repair; LV, left ventricular; SEVR, subendocardial viability ratio; TEVAR, thoracic endovascular aortic repair.

TEVAR may produce particularly important biomechanical effects because it covers the highly compliant proximal or descending thoracic aorta. Tzilalis et al. (2012) reported increased arterial stiffness and hypertension in young patients following thoracic stent‐grafting. In 64 patients undergoing TEVAR, Yamashita et al. (2021) reported that baPWV increased from 1851 ± 392 to 2047 ± 479 cm/s, with 58% demonstrating an increase of at least 100 cm/s. Older age, coronary artery disease and perioperative changes in heart rate and systolic blood pressure were associated with the measured change. Because this study assessed baPWV, its findings should not be interpreted as a direct measurement of isolated aortic stiffness.

Procedure‐related characteristics may modify the response. Hori et al. (2021) found that treatment length and device selection, but not treatment site alone, were associated with changes in baPWV after TEVAR. In a long‐term study of 26 patients treated with TEVAR for blunt thoracic aortic injury, Guala et al. (2024) reported hypertension in 65%, abnormal nocturnal blood‐pressure drop in 54% and high cfPWV in 23% at a mean follow‐up of approximately 10 years. New‐onset hypertension was associated with a more proximal landing zone and greater distal oversizing, while high cfPWV was associated with treatment length and premature arterial ageing. These observational associations identify plausible procedural modifiers but do not establish that altering one variable will improve outcomes.

Experimental studies provide supporting mechanistic evidence. Nauta et al. (2016, 2017) demonstrated reduced longitudinal and radial deformation after TEVAR in *ex vivo* models, with strain discontinuities between treated and native aorta. De Beaufort et al. (2017) found progressive increases in pulse‐wave velocity as thoracic stent‐graft coverage was extended in an *ex vivo* porcine model. These findings support the concept of compliance mismatch but should not be interpreted as direct clinical evidence that TEVAR causes subsequent dilatation, dissection or cardiovascular events.

Emerging 4D‐flow CMR suggests that the mechanical response is segment specific rather than uniform. Hauguel et al. (2026) observed altered secondary‐flow patterns after descending TEVAR and an increase in pulse‐wave velocity within the stented segment alongside a different response upstream. Such findings explain why regional and local measurements may provide distinct but complementary information.

Dedicated stiffness data following fenestrated or branched endovascular repair of thoracoabdominal aneurysms remain scarce. Longer aortic coverage and multiple bridging stents plausibly create a larger compliance discontinuity, but this hypothesis has not been confirmed by robust longitudinal clinical evidence. Extrapolation from standard EVAR or TEVAR should therefore be explicit and cautious.

Overall, endovascular repair increases arterial stiffness in many, but not all, cohorts. Current evidence does not establish a stiffness threshold that should influence procedural selection or mandate additional clinical surveillance. The principal research priorities are to determine which patient and procedural characteristics predict clinically important changes and whether those changes contribute independently to hypertension, ventricular remodelling or cardiovascular events.

### 3.3 Open surgical repair: Where does it stand?

OSR removes or excludes diseased aortic tissue and replaces it with a prosthetic interposition graft. Although an open graft does not contain the metallic framework of an endograft, commonly used polyester and expanded polytetrafluoroethylene grafts remain substantially less compliant than native aorta. Open repair should therefore not be regarded as a mechanically neutral or compliant control. Its effect depends on graft material and length, anatomical location, reconstruction configuration and the mechanical properties of the remaining native aorta.

Clinical findings after open abdominal aortic repair are heterogeneous (Table 2). In a prospective pilot study of 34 patients, Gray et al. (2016) reported a mean reduction of 0.2 in the measured pulse‐wave velocity following open repair compared with an increase of 3.3 following EVAR; however, only 15 patients underwent open repair, and the study was not powered to establish biomechanical superiority. By contrast, Paraskevas et al. (2009) reported that cfPWV increased from 7.84 ± 1.85 to 10.08 ± 1.57 m/s 6 months after open repair in 24 patients. Importantly, that study did not demonstrate that the increase reduced cardiovascular or rupture risk; the authors stated that its clinical significance remained to be established.

**TABLE 2:** Evidence following open repair and direct open‐endovascular comparisons.

Study	Population and repair	Exact assessment and timing	Principal finding	Interpretation / limitation
Paraskevas et al. (2009)	24; open infrarenal AAA repair	cfPWV; pre‐repair and 6 months	7.84 ± 1.85 to 10.08 ± 1.57 m/s	No comparator; clinical meaning of increase unproven
Lantelme et al. (2009)	50; 39 OSR and 11 stent‐graft	cfPWV, carotid‐radial PWV and AIx; median 47 days	cfPWV +1.0 m/s overall; no significant prosthesis‐type effect; AIx diverged by repair	Small endovascular subgroup; stiffness and wave reflection differed
Gray et al. (2016)	34; 15 OSR and 19 EVAR	Ultrasound‐derived PWV; pre‐ and post‐repair	Mean change ‐0.2 after OSR versus +3.3 after EVAR; postoperative value lower after OSR	Prospective pilot; small sample; reported units require caution
Mylonas et al. (2021)	110; 54 OSR and 56 EVAR	CAVI; baseline, 48 h and 6 months	Early increase in both; return towards baseline after OSR, persistent elevation after EVAR	CAVI includes peripheral arterial pathway
Marketou et al. (2021)	73; 12 OSR and 61 EVAR	cfPWV and LV GLS; baseline, 1 and 6 months	cfPWV increased and GLS deteriorated; changes correlated; no repair‐group interaction	All men; small OSR group; cardiac association not proof of causation
de Kort et al. (2025)	15 *ex vivo* porcine aortas; open descending interposition graft	Regional PWV and pressures before/after 10‐cm polyester graft	PWV increased by approximately 10%; pulse pressure increased	Experimental model; not directly generalisable to patients

Abbreviations: AAA, abdominal aortic aneurysm; AIx, augmentation index; CAVI, cardio‐ankle vascular index; cfPWV, carotid‐femoral pulse‐wave velocity; EVAR, endovascular aneurysm repair; GLS, global longitudinal strain; LV, left ventricular; OSR, open surgical repair; PWV, pulse‐wave velocity.

Lantelme et al. (2009) evaluated 50 patients, including 39 treated with an open graft prosthesis and 11 with a stent‐graft. cfPWV increased by approximately 1.0 m/s across the cohort, but the effect of prosthesis type on cfPWV was not significant. Augmentation index increased after stent‐grafting and decreased after open graft placement, illustrating that stiffness and wave‐reflection indices may respond differently and should not be treated as interchangeable outcomes.

Mylonas et al. (2021) assessed CAVI in 110 patients undergoing open repair or EVAR. CAVI increased in both groups in the immediate postoperative period. At 6 months, values returned towards baseline following open repair but remained elevated following EVAR. Because CAVI incorporates the aortic and peripheral arterial pathways, these findings support a difference in global arterial response but do not provide a direct segmental measurement of graft compliance.

More recent experimental evidence also challenges the assumption that open replacement preserves native biomechanics. In an *ex vivo* porcine model, replacement of a 10‐cm descending thoracic aortic segment with a polyester graft increased pulse‐wave velocity by approximately 10% and increased pulse pressure (de Kort et al., 2025). In an *ex vivo* human model, Yusefi et al. (2025) directly compared TEVAR with open arch replacement, further demonstrating that both forms of prosthetic repair can disturb native aortic mechanics. Experimental findings cannot be directly extrapolated to patients, but they reinforce the relevance of compliance mismatch to open as well as endovascular repair.

Taken together, open repair may cause a smaller or less persistent increase in some stiffness measurements than endovascular repair, but the evidence is neither uniform nor sufficiently robust to establish open repair as biomechanically superior. Comparisons remain limited by small cohorts, different measurement pathways, short follow‐up and non‐random treatment allocation. Evidence following open thoracoabdominal replacement is particularly limited, despite the potentially large length of prosthetic aorta involved.

### 3.4 Endografts: Innovation and technology

Historically, aortic endografts have been designed primarily to achieve exclusion, fixation, sealing, conformability and procedural durability. These remain the essential determinants of device performance. An emerging design objective is to reduce the compliance mismatch between the prosthesis and native aorta without compromising radial strength or sealing. The available evidence, however, does not yet support selection of a commercial device according to its presumed effect on arterial stiffness.

#### 3.4.1 Woven polyester versus expanded polytetrafluoroethylene

Polyester and expanded polytetrafluoroethylene (ePTFE) differ in their mechanical and inflammatory properties, but clinical comparisons cannot isolate the effect of fabric from the stent framework, graft geometry, fixation system and implantation technique. In a non‐randomised study of 118 men undergoing EVAR, Kadoglou et al. (2014) reported increased cfPWV in both fabric groups at 12 months, with a larger increase among patients receiving polyester‐covered devices than among those receiving ePTFE‐covered devices (*P* = 0.033). The observational design and use of different commercial devices mean that the findings should be viewed as hypothesis generating rather than evidence that polyester is intrinsically inferior.

Van Herwaarden et al. (2006) assessed only 11 patients using dynamic magnetic resonance angiography. Patients receiving an ePTFE Excluder device demonstrated higher local aneurysm‐neck stiffness and elastic modulus than those receiving a polyester Talent device. This does not directly contradict the cfPWV findings of Kadoglou et al., because the studies examined different mechanical endpoints: one measured regional cfPWV, whereas the other assessed local neck mechanics. The small samples, older device generations and non‐randomised allocation further preclude a clinically reliable comparison between fabrics.

Fabric‐related inflammatory responses should also be distinguished from mechanical stiffness. Evidence that one material produces a greater perioperative inflammatory response does not establish that the same material causes greater long‐term aortic stiffness or worse cardiovascular outcomes (Baek et al., 2015). Current evidence therefore does not support selecting polyester or ePTFE solely to minimise arterial stiffness.

#### 3.4.2 Endoskeleton versus exoskeleton

Stent‐frame position may influence composite device compliance, but the clinical evidence remains limited. Hori et al. (2020) compared 60 patients receiving endoskeleton devices with 26 receiving exoskeleton devices for aortic arch aneurysms. baPWV increased significantly in the exoskeleton group and continued to increase during a median follow‐up of 32 months, whereas no significant change was observed in the endoskeleton group. Long‐term ventricular and cardiovascular outcomes also differed between groups. However, this was a non‐randomised observational comparison, and residual differences in patient characteristics, device selection and procedural anatomy cannot be excluded.

Other cohorts have not consistently identified stent‐frame category as an independent determinant. Yamashita et al. (2021), for example, found that age, coronary artery disease and perioperative haemodynamic changes were more clearly associated with changes in baPWV than device category. Consequently, endoskeleton designs may represent a compliance‐preserving hypothesis, but the evidence is insufficient to conclude that they reproduce native aortic mechanics or improve clinical outcomes.

#### 3.4.3 Commercial devices and emerging compliance‐aware design

Available studies do not permit a reliable ranking of commercial endografts according to arterial stiffness (Table 3). Fabric, stent alloy, frame position, radial force, graft length, diameter, oversizing and fixation mechanism vary simultaneously among devices. Device selection is also influenced by anatomy and pathology, producing substantial confounding by indication. Comparisons involving the Excluder, Talent, Endurant, Zenith and other devices should therefore be interpreted as individual device observations rather than evidence of a class effect or commercial hierarchy.

**TABLE 3:** Graft material, frame configuration and emerging compliance‐aware technology.

Study	Material / device comparison	Exact endpoint	Principal finding	Interpretation / limitation
van Herwaarden et al. (2006)	Excluder (ePTFE) versus Talent (polyester); *n* = 11	Local aneurysm‐neck Ep and beta by dynamic MRA	Excluder showed higher local stiffness indices than Talent	Very small, non‐randomised cohort; local endpoint, not cfPWV
Kadoglou et al. (2014)	Polyester *n* = 72 versus ePTFE *n* = 46; EVAR	Regional cfPWV at 12 months	Increase in both; larger with polyester (+4.12 ± 0.33 versus +2.82 ± 0.25 m/s)	Fabric confounded by complete device design
Morris et al. (2016)	Four commercial stent‐grafts; experimental flow model	Device/wall compliance mismatch	All devices altered compliance; magnitude varied by design	Bench model; cannot rank clinical outcomes
Hori et al. (2020)	Endoskeleton *n* = 60 versus exoskeleton *n* = 26; TEVAR	baPWV and echocardiography	Progressive baPWV rise and cardiac changes in exoskeleton group	Non‐randomised; anatomy and device choice may confound
Girardin et al. (2024)	Patient‐specific virtual grafting in TBAD	Compliant CFD; pressure and energy loss	Longer/stiffer grafts increased pressure and energy loss; compliance matching reduced pulse pressure	Single‐case in silico evidence
Shahbad et al. (2026)	Conventional polyester versus experimental nanofibrous stent‐graft	Bench‐top PWV, pulse pressure and distensibility	Experimental compliant graft remained closer to native mechanics	Preclinical device; no patient outcomes
Kamenskiy et al. (2026)	Conventional versus Windkessel‐preserving stent‐graft; porcine TEVAR	Aortic pulsatility and LV remodelling	Compliant graft preserved pulsatility and attenuated LV remodelling	Preclinical; requires human validation

Abbreviations: baPWV, brachial‐ankle pulse‐wave velocity; CFD, computational fluid dynamics; cfPWV, carotid‐femoral pulse‐wave velocity; Ep, pressure‐strain elastic modulus; ePTFE, expanded polytetrafluoroethylene; LV, left ventricular; MRA, magnetic resonance angiography; PWV, pulse‐wave velocity; TBAD, type B aortic dissection; TEVAR, thoracic endovascular aortic repair.

This limitation is particularly apparent when regional and local measurements are compared. Van Herwaarden et al. (2006) reported differences in locally measured aneurysm‐neck stiffness between Excluder and Talent devices, whereas Hori et al. (2020) evaluated baPWV and longer‐term cardiac outcomes in patients with thoracic devices. Such studies cannot be combined into the conclusion that one commercial brand is more or less compliant.

Emerging research is instead examining the mechanics of the complete graft–aorta system. Patient‐specific computational modelling suggests that longer and stiffer grafts increase pulse pressure and energy loss, whereas matching graft compliance to the native aorta may attenuate these effects (Girardin et al., 2024). Bench‐top studies of more compliant nanofibrous graft materials have similarly reported preservation of distensibility and smaller increases in pulse‐wave velocity than conventional polyester stent‐grafts (Shahbad et al., 2026). In a porcine TEVAR model, an experimental Windkessel‐preserving stent‐graft maintained aortic pulsatility and attenuated left‐ventricular remodelling compared with a conventional device (Kamenskiy et al., 2026).

These developments illustrate the transition from devices optimised primarily for structural exclusion towards compliance‐aware or biomimetic design. Nevertheless, the evidence remains computational, bench‐top or preclinical. No compliance‐matched experimental graft has demonstrated superior long‐term cardiovascular outcomes in a clinical comparative trial, and current commercial‐device selection should remain based on established anatomical, procedural and durability considerations.

### 3.5 Impact of patient characteristics

Interpretation of arterial stiffness after aortic repair requires separation of the treatment effect from the patient's baseline vascular phenotype. Age, mean arterial pressure, heart rate, hypertension, diabetes, chronic kidney disease, smoking, systemic inflammation and pre‐existing atherosclerotic disease are associated with arterial stiffness independently of intervention (McEniery et al., 2010; Mitchell et al., 2004). Long‐term blood‐pressure variability may additionally contribute to progression of arterial stiffness (Tedla et al., 2017). These variables may confound comparisons between OSR and endovascular repair and should be measured and adjusted for in longitudinal studies.

Sex‐related differences have been demonstrated in general‐population studies, with women showing a steeper age‐related decline in aortic distensibility after midlife in some cohorts (Nethononda et al., 2015; Tomiyama et al., 2003). However, evidence for a sex‐specific response to aortic graft implantation remains limited. The general association between menopause and arterial stiffness should not be interpreted as proof that women experience a greater repair‐induced increase or require a different graft strategy.

Procedural characteristics may be as important as demographic factors. Emerging evidence identifies treatment length, landing‐zone location, oversizing and the proportion of compliant native aorta covered as potential determinants of the postoperative haemodynamic response (Guala et al., 2024; Hori et al., 2021). Baseline aortic disease also matters: an aneurysmal, dissected or traumatically injured aorta does not share a single mechanical phenotype. Future studies should therefore report baseline cfPWV and haemodynamic variables alongside detailed anatomical and device characteristics rather than attributing postoperative differences to repair type alone.

### 3.6 Cardiac outcomes

Aortic stiffness has been associated with cardiovascular events and mortality across multiple populations. In a meta‐analysis of 15,877 participants, each 1 m/s increase in aortic pulse‐wave velocity was associated with adjusted increases of approximately 14% in total cardiovascular events, 15% in cardiovascular mortality and 15% in all‐cause mortality (Vlachopoulos et al., 2010). An individual‐participant meta‐analysis of 17,635 participants similarly found that aortic pulse‐wave velocity improved cardiovascular‐risk prediction beyond conventional risk factors (Ben‐Shlomo et al., 2014). These associations provide biological and prognostic context but do not establish that an increase caused by an aortic graft produces an equivalent increment in clinical risk.

Repair‐specific longitudinal evidence is more limited. Takeda et al. (2014) reported increased baPWV after endovascular aortic repair accompanied by increases in left‐ventricular mass index and left‐atrial volume index. These findings suggest a possible relationship between altered arterial load and cardiac remodelling, but baPWV incorporates peripheral arteries and the study did not establish the endograft as the sole cause. Left‐ventricular mass and atrial volume are therefore downstream outcomes rather than measurements of aortic stiffness.

More direct evidence was provided by Marketou et al. (2021), who prospectively assessed 73 men undergoing open repair (n = 12) or EVAR (n = 61). cfPWV increased at 1 month and remained elevated at 6 months (*P* = 0.007), while left‐ventricular GLS deteriorated over the same period (*P *< 0.001). Changes in GLS correlated with changes in cfPWV; however, there was no significant group effect or interaction between open and endovascular repair. The study supports an association between postoperative stiffness and early myocardial dysfunction but does not demonstrate that one repair approach produces greater cardiac impairment.

Long‐term evidence is beginning to emerge after TEVAR in younger patients. In addition to the findings of Guala et al. (2024), Vallerio et al. (2019) and Kamenskiy et al. (2021) reported hypertension, increased left‐ventricular mass or off‐target aortic remodelling after TEVAR for blunt thoracic aortic injury. In a retrospective comparison of 50 TEVAR and 50 OSR patients, Yuan et al. (2025) found no clear unadjusted difference in cardiac remodelling at 12–36 months, although adjusted analysis (for patient demographic factors of age, sex, and body surface area) suggested a modestly greater antihypertensive medication burden after TEVAR (*P* = 0.044). No significant differences were reported between the two groups in left ventricular ejection fraction and left ventricular hypertrophy. These results are clinically relevant but remain susceptible to confounding and use medication burden as an indirect haemodynamic outcome.

Separately, the meta‐analysis by Chen et al. (2026) found an increase in left‐ventricular mass index after endovascular repair, but again no significant change in left‐ventricular ejection fraction. The preservation of ejection fraction does not exclude subclinical myocardial effects, but high heterogeneity and the small size of the contributing studies limit causal interpretation. Evidence directly linking repair‐induced stiffness to major cardiovascular events remains insufficient.

Accordingly, routine aortic imaging, blood‐pressure management and cardiovascular‐risk reduction should continue according to established disease‐specific guidance. There is currently insufficient evidence to recommend additional clinical surveillance, a different repair approach or a particular device based on a postoperative stiffness threshold alone.

Future studies should adopt a standardised longitudinal framework. At minimum, cfPWV should be assessed using the same validated technique before repair, during an early postoperative interval and at 6–12 months. Studies should concurrently report brachial and central blood pressure where available, mean arterial pressure, heart rate, antihypertensive medication burden and relevant comorbidities. Procedural reporting should include pathology, treated segment, graft length, landing zones, oversizing, fabric and stent‐frame configuration. Echocardiographic outcomes should include left‐ventricular mass, diastolic function and GLS, while segmental CMR or 4D‐flow assessment may be incorporated into mechanistic substudies. Longer‐term endpoints should include incident hypertension, escalation of antihypertensive therapy, ventricular remodelling, cardiovascular events, aortic remodelling and reintervention.

## 4 CONCLUSION

cfPWV is the accepted reference non‐invasive measure of regional aortic stiffness. The central methodological question is therefore not which technique should replace cfPWV, but how a standardised regional measure can be combined with local imaging and downstream cardiovascular outcomes to characterise the effects of aortic repair. Established evidence indicates that prosthetic aortic repair can alter arterial mechanics. Increases in stiffness are reported more consistently after EVAR and TEVAR, although open graft replacement should not be regarded as a mechanically neutral control. The magnitude and distribution of change appear to depend on the treated segment, graft length, landing zone, oversizing, baseline vascular phenotype and measurement pathway. Current comparisons among graft fabrics, stent configurations and commercial devices are too small and heterogeneous to support a clinically reliable ranking. Emerging 4D‐flow imaging, patient‐specific modelling, and compliant or biomimetic graft materials illustrate the transition from structural repair alone towards biomechanical compatibility. These approaches remain investigational and have not yet demonstrated improved long‐term clinical outcomes. Future research should use standardised preoperative and longitudinal cfPWV, blood‐pressure and cardiac assessments; report procedural and device characteristics in sufficient detail; and relate biomechanical changes to incident hypertension, ventricular remodelling, cardiovascular events and aortic outcomes. Until such evidence is available, repair strategy and device selection should remain based on established anatomical, procedural and clinical considerations.

## AUTHOR CONTRIBUTIONS

Mohamad Bashir, Idhrees Mohammed, Ian M. Williams and Damian M. Bailey contributed to the conception and design of the work and to drafting or critically revising the manuscript for important intellectual content. Thurkga Moothathamby, Matti Jubouri, Fatima Kayali, Sakshi Roy and Subham Roy contributed to the acquisition, analysis or interpretation of literature and to drafting or critically revising the manuscript. All authors have read and approved the final version of this manuscript and agree to be accountable for all aspects of the work in ensuring that questions related to the accuracy or integrity of any part of the work are appropriately investigated and resolved. All persons designated as authors qualify for authorship, and all those who qualify for authorship are listed.

## CONFLICT OF INTEREST

D.M.B. is Editor‐in‐Chief of *Experimental Physiology*, Chair of the Life Sciences Working Group and a member of the Human Spaceflight and Exploration Science Advisory Committee to the European Space Agency and of advisory committees to the UK and Swedish space agencies. D.M.B. is also affiliated with Bexorg, Inc. (USA), which focuses on the technological development of biomarkers of cerebral bioenergetic function and structural injury. The remaining authors declare no competing interests.

## FUNDING INFORMATION

Damian M. Bailey is a Royal Society Wolfson Research Fellow (WM170007). No specific funding was received for the preparation of this review.

## GENERATIVE AI STATEMENT

OpenAI ChatGPT 5.6 was used solely to assist with the formatting and presentation of the manuscript tables. It was not used to generate scientific content, analyse data, interpret findings or formulate conclusions. All table content and formatting were independently reviewed and approved by the authors.

## Data Availability

All evidence discussed in this narrative review is available in the cited published literature.
